# COVID-19 and mental health: A systematic review of international medical student surveys

**DOI:** 10.3389/fpsyg.2022.1028559

**Published:** 2022-11-25

**Authors:** Dean C. Paz, Manav Singh Bains, Morgan L. Zueger, Varasiddimounish R. Bandi, Victor Y. Kuo, Kyle Cook, Rebecca Ryznar

**Affiliations:** ^1^College of Osteopathic Medicine, Rocky Vista University, Parker, CO, United States; ^2^Department of Biomedical Sciences, Rocky Vista University, Parker, CO, United States

**Keywords:** medical students, COVID-19, anxiety, depression, mental health, mood, SARS-CoV-2, anxiety disorder

## Abstract

**Background:**

The medical school curriculum has imposed psychological stressors on students worldwide, some of which may induce feelings of increased depression and anxiety. Meanwhile, the COVID-19 pandemic has exacerbated the feelings of stress, depression, and anxiety that a portion of medical students experience in their daily life. The aim of this systematic review is to gather concrete data from medical schools around the globe, and further evaluate how the COVID-19 pandemic has impacted medical students’ mental health.

**Materials and methods:**

Systematic search of data from PubMed, EMBASE, psycINFO, MEDLINE Complete, and Global Health for studies conducted between December 2019 and July 2021 was conducted. Data from 47 different surveys of medical students from various medical institutions throughout the world were included in this review. These surveys, administered in the form of questionnaires that utilized rating scales, measured anxiety, depression, and stress levels in medical students amidst the COVID-19 outbreak.

**Results:**

The COVID-19 outbreak was found to have negative effects on medical students, most notably causing emotional and behavioral changes and detrimental mental health impacts. Higher levels of stress, depression, and anxiety have been found in medical students since the outbreak.

**Conclusion:**

This systematic review highlights the sustained high prevalence of moderate depression, anxiety and stress among medical students during the COVID-19 pandemic. Appropriate support and research on which interventions could mitigate these risks is essential in order to ensure that future physicians are properly cared for, and ultimately have the adequate tools needed to provide high quality and empathetic care to future patients.

## Introduction

In November 2019, the first SARS-CoV-2 outbreak hit Wuhan, China, which became the epicenter of what would become the worst pandemic of modern society, and the first major one that healthcare professionals and civilians had encountered. This Corona virus disease showed to be an incredibly infectious and debilitating agent that impacted people of all ages around the world. On March 11, 2020, the World Health Organization declared the Corona virus disease a global pandemic (World Health Organization [WHO], 2020). As of August 24, 2021, the World Health Organization has documented 212 million COVID-19 cases, and 4.4 million deaths attributed to this pandemic outbreak (World Health Organization [WHO], 2022). This COVID-19 pandemic has heavily impacted the lives of many people worldwide who are suffering from illness, losing loved ones, or struggling to cope with the new reality of isolation and social distancing. The pandemic has shown the importance of being grateful for the relationships we have, and has proven how valuable mental health truly is. A significant majority of the studies we found focus on mental health of global medical students, and there is a major gap in the literature discussing how the COVID-19 pandemic has impacted medical student mental health in the United States. Despite the emergence of the vaccines and their important role in the decrease in number and severity of cases, it is of utmost importance that we continue to explore how the COVID-19 pandemic impacted the next generation of physicians as they progress in their medical journey.

Significant changes were made to the traditional medical school curriculum throughout the world, leaving many overwhelmed about their belief in their success and furthering the challenges already imposed on medical students. Students have been forced to amend their learning and studying styles, while academic institutions have similarly been strained with the responsibility of changing their previously well-established education curriculum and mode of delivery (Al Samaraee, 2020). Medical education has been notoriously considered as one of the foremost rigorous and demanding academic journeys, with great potential to induce outstanding psychological stress in a medical student (Backović et al., 2013; Awad et al., 2019; Quek et al., 2019). Medical institutions globally have taken significant consideration into documenting how their students have been impacted, but institutions in the United States have been less proactive in the process. As current medical students, we will be focusing our paper on how the overall experience of medical education, with all the pre-listed restrictions and negative effects faced during the pandemic, have affected the psyche of the medical student population.

The rigor, intensity, and challenges that come with online learning during medical school has inevitably led to social isolation, maladaptive behaviors, and feelings of decreased confidence (Alsoufi et al., 2020; Mheidly et al., 2020; Zis et al., 2021). This begs the questions of whether the COVID-19 pandemic has exacerbated the mental health of medical students or had no impact. The primary aim of this systematic literature review is to identify the severity of mental health decline in medical students internationally, and provide updated estimates of the prevalence of depression, stress, and anxiety amongst medical students from various countries throughout the COVID-19 pandemic. Utilizing this data will aid in preparation for future life-changing events that may have similar impedances on medical students’ life. The data will ultimately emphasize the necessity in evaluations and treatments to mitigate mental health challenges, and will hopefully induce additional strategies that medical institutions can employ. We ultimately believe that medical students’ mental health during the COVID-19 pandemic has been negatively exacerbated, and that feelings of anxiety, depression, and stress are more prominent. In order to ensure that future physicians are able to provide adequate and competent care to their patients, it is imperative that institutions around the world develop strategies to improve these students’ mental health.

## Materials and methods

This systematic review was conducted in accordance with the Preferred Reporting Items for Systematic Reviews and Meta-Analysis (PRISMA) guidelines (Page et al., 2021a,b). The review was not registered and a review protocol was not prepared in a formal document, but is discussed thoroughly below. Review authors were unable to find a duplicate registered protocol and given the unique nature of the review question, review authors did not believe registration was necessary. Review authors have no competing interests to declare. There were no sources of financial or non-financial support for this review.

### Search strategy

Articles were retrieved through a systematic search of PubMed, EMBASE, psycINFO, MEDLINE Complete, and Global Health. Searches for each database were conducted on August 9th, 2021. The overarching search tool included our Rocky Vista University database, which encompasses the more specific databases previously listed. The same search string was used for each database.

The search string for each database was the following: (“medical student” or “medical students”) AND (“COVID-19” or “Corona virus” or “2019-nCOV” or “SARS-CoV-2” or “CoV-19”) AND (”anxiety” or “depression” or “stress” or “anxiety disorder” or “mental health” or “mood”).

### Inclusion and exclusion criteria

Studies were included if they met the following criteria: (1) presented original research (2) published in English since the outbreak of COVID-19 in December 2019; (3) assessed depression, anxiety or stress among medical students; (4) used an established or well-designed method to measure depression, anxiety or stress; (5) provided sufficient information to calculate prevalence of depression, anxiety or stress among health care workers (e.g., percentage or sample size and number). We excluded studies that focused on non-medical students (including undergraduate, graduate, or other health profession students; medical residents or doctors), discussion of medical students grouped with non-medical students, studies that did not involve measurements of anxiety, depression, or stress, studies that solely collected anxiety, depression, or stress data using questions with binary responses (for example, only reported anxiety data collected from the question, “did you experience anxiety during the pandemic?” with available answer responses “yes” or “no”), studies that did not involve study of impact of COVID-19, studies that did not contain concrete data, including published conference abstracts, and studies that were not in English.

### Study selection

Authors DP, MB, VB, VK, and MZ screened all titles and abstracts against the selection criteria. There was 99% agreement between reviewers. Full texts for selected studies were reviewed by DP, MB, VB, VK, and MZ. When the relevance of a study was unclear, the authors reread the full text of each study and discussed their disagreements on inclusion versus exclusion criterion until disagreements were resolved. Citations were generated by manual input into Zotero.

### Data collection

Data was independently and manually extracted by authors DP, MB, VB, VK, and KC and reviewed collectively by DP, MB, and VK. No study data requiring confirmation from authors was identified. The following data was extracted from included studies, with use of a standardized form: (1) Study Characteristics: country, population, sample method, sample size, response rate, gender, and age; (2) Outcome: reported statistical analysis of depression, anxiety, and stress levels from psychological scales, instruments used, related factors assessed, and relevant outcomes and results. Additional data collected by studies outside of the specified parameters were not reported. All reported effect measures for outcomes were drawn from study data and were not independently verified given the number of included studies and that all included studies were peer-reviewed. Reported data was not synthesized given the wide range of outcomes between studies and the goals of this review.

### Quality assessment

The studies included were assessed for risk of bias using the instrument from Agarwal et al. “Risk of Bias in Cross-Sectional Surveys of Attitudes and Practices.” Rather than giving us a single risk of bias for a single study, when addressing cross-sectional surveys, this instrument will help us provide risk of bias on a domain-by-domain basis, instead of one overall risk of bias rating and/or when the domains are inter-related with one another (Quek et al., 2019). This instrument was selected for its applicability to these review–all studies included is cross-sectional surveys–and its adaption from an established tool, the Cochrane risk-of-bias instrument for randomized trials (Cochrane methods Bias, 2022). As determined by the instrument, risk of bias was assessed across five different domains: (1) Is the source population representative of the population of interest? (2) Is the response rate adequate? (3) Is there little missing data? (4) Does the study have a pilot study? (5) Is there any evidence for the reliability and validity of the survey instrument? Each domain is rated on a four-point scale with 1 signifying “definitely yes” (low risk of bias) and 4 signifying “definitely no” (high risk of bias). Quality assessments for the studies included in this review can be found in Supplementary Appendix Table 3. Reported certainty assessments were drawn from study data and were not independently verified given the number of included studies and that all included studies were peer-reviewed.

## Results

### Introduction

**FIGURE 1:**
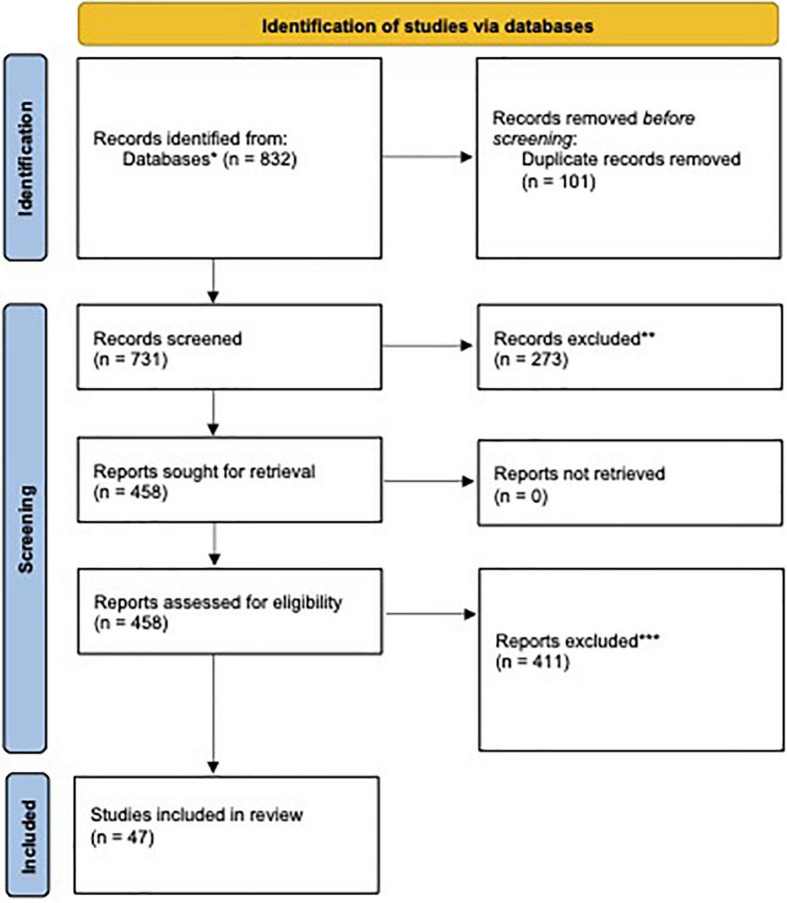
PRISMA 2020 flow diagram for new systematic reviews which included searches of databases and registers only. *Breakdown by source: PubMed (*n* = 220), EMBASE (*n* = 354), PsycINFO (*n* = 13), MEDLINE complete (*n* = 206), and global health (*n* = 39). **Exclusion was done manually by researchers. No automation tools were used. Reasons included (1) did not involve medical students, (2) did not study the impact of COVID-19, (3) not accessible in English, and (4) publications that were not full-text (i.e., conference abstracts). ***Reasons for exclusion included (1) did not involve measurements of anxiety, depression, or stress, (2) medical students were grouped with non-medical students, and (3) self-reported anxiety, depression, or stress.

The literature search identified 832 articles published between December 1st, 2019 and August 9th, 2021 (Figure 1). 101 duplicates were removed. 273 articles were removed during title and abstract screening, and 458 articles were subjected to full-text analysis and 411 articles were removed according to the inclusion/exclusion criteria described above. In total, 47 studies were included in this review. Analysis of 40 of the most relevant studies has been organized by country or region below. A breakdown of studies by country includes: Nine studies in China, one study in Australia, one Kazakhstan, three studies in Turkey, one study in Saudi Arabia, two studies in Germany, two studies in Japan, five studies in the United States of America, one study in Italy, one study in Nepal, one study in Libya, two studies in Canada, one study in Bangladesh, one study in Jordan, one study in Ireland, six studies in India, one study in Lebanon, two studies in Brazil, one study in Morocco, one study in Singapore, one study in Thailand, one study in Peru, one study in Pakistan, and one study in Russia. Study characteristics, prevalence data, and relevant results for all 47 included studies can be found in Supplementary Appendix Tables 1, 2.

Study quality varied by study. The most common drawbacks for studies were associated with limited response rate and small sample size. The majority of studies used reported data from cross-sectional surveys, which have certain inherent biases, as discussed below. Response rate was generally low, which is not unexpected for voluntary surveys. Data analyses were determined to be appropriate for all studies.

Across all studies and regions, COVID-19 was reported to have a nearly ubiquitous negative impact on medical student populations and was associated with increased levels of anxiety, stress, and depression. The following results are described based on geographical regions. This was done to compare how different articles varied and shared similarities amongst each other in specific regions because they share the underlying commonality of geography. Some countries had more papers published then others, hence why China and India had their own sections instead of being included in sections with other regionally similar countries. From a grand scale, this model allows for simplicity to compare different geographical regions amidst the differences in how national governments reacted to the pandemic. As a result, these differences in public policy are the basis for how medical schools were affected, and hence their students’ mental health as well.

### China

In our evaluation of various studies from China, COVID-19 was generally reported to have a negative impact on medical student mental health, most prominently by exacerbating students’ anxiety, depression, and stress levels. For example, a cross-sectional study on medical students from 3 Chinese medical universities was conducted to evaluate perceived stress, insomnia, and depression using the Perceived Stress Scale (PSS), Insomnia Severity Index (ISI), and Patient Health Questionnaire 9 (PHQ-9). Of 29,663 participants, perceived stress was significantly associated with depression and insomnia (Liu et al., 2021). Utilizing the Depression, Anxiety and Stress Scale (DASS-21), researchers in another study involving 1,041 participants detected that 26.8% of students in China had elevated levels of depression, 20.2% had elevated levels of anxiety, and 11.1% had elevated levels of stress (Zhang et al., 2021a). Fujian Medical University conducted a survey that assessed 906 medical students from 12 different medical colleges, revealing a positive relationship between mental stress and loneliness. This university also reported that the pandemic increased students’ mental stress due to fears of becoming infected with COVID-19 (Zheng et al., 2021). Meanwhile, Shantou University Medical College assessed two surveys conducted throughout the course of the pandemic, with 1,069 responses in the first study and 1,511 students in the second study, which revealed a rise in stress and anxiety levels between the two time-points (Zhang et al., 2021b). Additionally, three medical universities in Western China found that students had moderate to high levels of stress based on a distributed survey. Average item scores for stressors included in the survey were 2.72 for academics, 2.31 for psychosocial and 2.07 for health-related stressors, where a score of 1 indicated participants strongly disagreed with the item being a stressor and 5 indicated participants strongly agreed with the item being a stressor (Wang et al., 2021). At China Medical University, 666 participants were included in a research article where 64 (9.6%) of the participants reported depression from March to April 2020 (Zhao et al., 2021). Another cross sectional study that surveyed 5,982 medical students found that prevalence rates of mild to severe depressive symptoms were 35.2% and of anxiety symptoms were 22.8% (Yin et al., 2021). Students with low to medium levels of social support had higher risks of experiencing depressive or anxiety symptoms (Yin et al., 2021). Both COVID-19 exposure and living in areas with higher COVID-19 cases was positively associated with mild to severe depressive or anxiety symptoms (Yin et al., 2021). Lastly, medical students at Changzhi Medical College were surveyed for levels of anxiety and stress; the proportions of students with mild, moderate, and severe anxiety were 21.3, 2.7, and 0.9% respectively (Cao et al., 2020). Students that lived alone, were from rural areas, from families without a steady income, those who lived with parents, and those with an acquaintance infected with COVID-19 were all found to more likely experience severe anxiety (Cao et al., 2020). Also, worry about academic delays and influence of epidemic on daily-life was found to have moderate and positive correlation with levels of anxiety (Cao et al., 2020). A negative association between social support and anxiety symptoms experienced by students was found during the COVID-19 outbreak (Cao et al., 2020).

### India

Studies discussing anxiety, depression, and stress levels were also conducted among medical students in India. A study conducted in Chennai City surveyed 500 medical students between the ages of 17–20 (Saravanan et al., 2021). Men in the study had an anxiety prevalence rate of 69%, while only 31% of females experienced anxiety (Saravanan et al., 2021). Depression rates were 34% in men and 27% in females (Saravanan et al., 2021). Furthermore, researchers from a study conducted at the Medical College of North India of 331 students concluded that students’ uncertainty about the duration of the medical degree and exams was positively correlated with stress (Indian Journal of Public Health Research and Development, 2020). Lastly, a cross-sectional study was conducted on medical students in Bengaluru, India, which found that 23.2% of the students had depression, 20.7% had anxiety and 13% had stress ranging from mild to extremely severe (Shailaja et al., 2020). Students with adaptive attitudes and behaviors experienced less severe depression, anxiety, and stress than other students (Shailaja et al., 2020).

### Rest of Asia, excluding the Middle East

Studies were also administered in other parts of Asia, including Pakistan, Japan, Bangladesh, Kazakhstan, Nepal, and Singapore. At Harian Medical College in Pakistan, a study of 233 students showed a significant decrease in the level of depression and an increase in the level of anxiety and stress among medical students (Masud et al., 2021). At the Okayama University School of Medicine in Japan, a survey of 473 medical students found that students’ mental health status significantly worsened after the Japanese State of Emergency regarding the pandemic was implemented (Nishimura et al., 2021). 29.8% of these students had concerns about transitioning to an online learning format, which was correlated with generalized anxiety and depression (Zhao et al., 2021). A cross-sectional study from Showa University School of Medicine furthermore showed a significant degree of psychological distress among 28.5% of the students (Arima et al., 2020). Researchers in Nepal surveyed various medical colleges using the Hospital Anxiety and Depression Scale (HADS), demonstrating a positive correlation between depression and anxiety levels in students (Risal et al., 2020). A similar cross-sectional study in Bangladesh administered HADS to 425 medical students, indicating that 65.9% of medical students had varying levels of anxiety: 27.3% mild, 26.8% moderate, and 11.8% severe (Safa et al., 2021). Additionally, 49.9% of the medical students were found to have varying degrees of depressive symptoms, with 3.3% of surveyed students reporting severe depressive symptoms (Safa et al., 2021). Students who had significant fears of becoming infected with the virus were at higher risk for anxiety and depression by 3.5 and 2.7 fold, respectively (Safa et al., 2021). A questionnaire-based cross-sectional study of medical students in Kazakhstan at Astana Medical University analyzed the impact of the transition to online learning. Interestingly, the study revealed that depression and anxiety decreased after transitioning from traditional learning to online learning (Bolatov et al., 2021). In addition, students that had academic difficulties or who lived alone during the quarantine were more prone to depression and anxiety (Bolatov et al., 2021). Lastly, there was an online survey conducted among medical students in Singapore during the COVID-19 lockdown period demonstrated that students with lower perceived susceptibility to infection had a lower anxiety score (Koh et al., 2021).

### Middle East

Relevant studies conducted in the Middle East surveyed medical student populations in Turkey, Saudi Arabia, Jordan, Iran, and Lebanon. A cross-sectional survey study was conducted of medical students at Bezmialem Vakif University in Istanbul, Turkey. Of the participants, GAD-7 scores showed that 19.7% were experiencing severe anxiety, 17.4% moderate anxiety and 37.1% mild anxiety (Bilgi et al., 2021). PHQ-9 scores revealed that 21.9% were experiencing moderate to severe depression, and 23% mild depression (Bilgi et al., 2021). In a separate online survey questionnaire, students at the Faculty of Medicine of Yeni Yüzyıl University in Istanbul were found to have increased anxiety and distress as determined respectively by PSS and Impacts Events Scale-Revised (IES-R) scores (Torun and Torun, 2020). Mean scores were higher in women than men (Torun and Torun, 2020). A survey of 553 medical students at six medical schools in Jordan found that COVID-19 negatively impacted the stress levels of 56.2% of students, as assessed by Kessler’s psychological stress scale (K10) (Seetan et al., 2021). Stress levels of students at the King Saud University College of Medicine (KSU) in Saudi Arabia were also assessed using K10. Overall stress levels were found to be significantly higher in female medical students and 3rd year medical students (Abdulghani et al., 2020). In addition, 22.3% of the students reported experiencing severe stress related to online learning (Abdulghani et al., 2020). Lastly, a descriptive survey study was conducted on medical students at the American University of Beirut Faculty of Medicine (AUBFM) in Lebanon. The majority reported that they felt more stressed after shifting to online classes and would be willing to go back to on-campus classes (Bachir et al., 2021).

### South America

Studies of medical student populations in Brazil and Peru were also analyzed as a part of this review. Students at Jundiai Medical School in Brazil reported high levels of stress during the COVID-19 pandemic, with first-year students reporting the most severe levels of stress. The survey also assessed HADS scores, which were found to have a negative correlation with year in school (Perissotto et al., 2021). A separate study conducted at Jundiai Medical School also found an inverse relationship between class year and depression, as first year medical students had the highest prevalence rates of depression (Miskulin et al., 2020). In Peru, 1,238 medical students were recruited to participate in a study, which reported that 74, 57, and 65% of the participants experienced depressive, anxious, and distress symptoms, respectively (Huarcaya-Victoria et al., 2021).

### North America

Studies conducted in the US and Canada were included in this review. A large study involving 1,428 medical students from 40 U.S. medical schools surveyed anxiety and depression using GAD-7 and PHQ-9; 30.6 and 24.3% screened positively for anxiety and depression, respectively (Halperin et al., 2021). Median GAD-7 and PHQ-9 scores were higher for females and preclinical students and median GAD-7 scores were higher among those with acquaintances diagnosed with COVID-19 (Halperin et al., 2021). Next, a cross-sectional survey was administered to medical students in clinical training at the University of California San Francisco School of Medicine, the University of California Irvine School of Medicine, Tulane University School of Medicine, the University of Illinois College of Medicine, the Ohio State University College of Medicine, and Zucker School of Medicine during the initial peak of the pandemic, April-May 2020. This investigation disclosed that 84.1% of the students reported increased levels of stress and anxiety due to the pandemic (Lee et al., 2021). Furthermore, according to GAD-7 scores, 34.3% showed mild, 16.1% were moderate, and 9.5% had severe anxiety symptoms (Lee et al., 2021). Female students were more likely to experience anxiety (Lee et al., 2021). In a separate survey involving students from these medical schools, students reported that the pandemic moderately affected their stress and anxiety levels with 84.1% of the participants reporting feeling “at least somewhat anxious” (Harries et al., 2021). In another study, a nationwide online survey was administered to medical students throughout the U.S. Of 852 participants, 66.1% experienced mild to severe anxiety (Guo et al., 2021). Mean PSS scores were highest among second through fourth year medical students (Guo et al., 2021). Students with preexisting mental health conditions had significantly higher stress and anxiety scores and students attributed the increased stress to COVID-19 (Guo et al., 2021). The last U.S. cross-sectional study analyzed in this review revealed that medical students reported higher levels of stress, anxiety, and depression than other graduate students (Nikolis et al., 2021). In a survey of 10 Canadian medical schools, 45% of surveyed medical students reported higher levels of stress than usual (Abbas et al., 2020). A different Canadian medical school reported that 58% of surveyed students felt more depressed and students with a prior history of depression or anxiety reported increased symptoms of anxiety and depression (ElHawary et al., 2021).

### Europe

Surveys were also conducted in the United Kingdom, Italy, and Germany. At the University College Dublin School of Medicine, 54.5% of students reported increased levels of stress ranging from moderate to extreme (O’Byrne et al., 2021). High levels of stress were more prevalent in females and international students (O’Byrne et al., 2021). At Magna Graecia University School of Medicine in Italy, 354 students were surveyed and 48.9% of the participants indicated an increased anxiety state (Abenavoli et al., 2021). In another study, a cross-sectional online survey was conducted on medical students at University Medical Center Hamburg-Eppendorf, Germany. Students were found to be experiencing “clinically unproblematic” levels of anxiety and depression symptoms, as assessed through Patient Health Questionnaire-4 (PHQ-4) scores (Guse et al., 2020). A final study from Germany reported that distress levels were found to be high and significantly correlated with academic context but notably, not with their private lives (Loda et al., 2020).

### Australia and Africa

Finally, three studies were included from Australia and Africa. In Australia, 297 students participated in a study using K10 to measure psychological distress (Lyons et al., 2020). The study reported a mean K10 score of 20.6, indicating moderate psychological distress (Lyons et al., 2020). In Morocco, the mental health of medical students throughout the country was assessed using PHQ-9, GAD-7, and K10. Out of 549 students, 62.3 and 74.6% screened positively for symptoms of anxiety and depression, respectively (Essangri et al., 2021). In a study involving 2,430 students from 15 medical schools in Libya, 64.5% of the students were determined to have varying degrees of anxiety according to GAD-7 scores (Elhadi et al., 2020). 37.5, 16, and 11% of students were determined to have mild, moderate and severe anxiety, respectively (Elhadi et al., 2020). Anxiety was significantly associated with living status and internal displacement (Elhadi et al., 2020). Furthermore, 21.6% of students had a PHQ-9 score indicating moderate to severe depression and 88% of the students scored positively for mild depression (Elhadi et al., 2020).

## Discussion

As we had asked the question of whether the COVID-19 pandemic had exacerbated the mental health of medical students or had no impact, the literature review was conducted to better understand the severity of mental health decline in medical students internationally, and provide updated estimates of the prevalence of depression, stress, and anxiety amongst medical students from various countries throughout the COVID-19 pandemic. After gathering the data and finalizing our results, we ultimately have found that medical students’ mental health during the COVID-19 pandemic has certainly been negatively exacerbated, and that feelings of anxiety, depression, and stress are more prominent.

In a meta-analysis conducted before the pandemic involving 40,348 medical students across the globe, researchers found the prevalence of anxiety to be 33.8%–a rate higher than that of the general population (Quek et al., 2019). Many factors can explain this high rate, including the financial burden, sleep deprivation, overwhelming workload, and lack of free time to find adequate methods to cope with chronic stress (Quek et al., 2019). In addition to anxiety, researchers have found higher rates of depression in medical students compared with the general public (Abenavoli et al., 2021). In an analysis of 167 cross-sectional studies, it was reported that the rate of depression among medical students was 27.2%, and of suicidal ideation was 11.1% (Liu et al., 2020). It was also discovered that among those who screened positive for depression, only 15.7% of medical students sought professional treatment (Liu et al., 2020). Anxiety and depression were found among students of all levels of education. Both studies did not find statistical significance of anxiety or depression among medical students and non-medical students, although the non-medical student groups often included those in professional or graduate programs, which may indicate that anxiety and depression are a consequence of the stressors of higher education in general. Nonetheless, these studies show that medical students experience higher rates of anxiety and depression, at baseline, compared to the general public. This is important to note in order to discuss the burden of the COVID-19 pandemic on medical students.

This systematic review highlights the implications of a global pandemic on medical student mental health. In this evaluation, COVID-19 was generally reported to have a negative impact on medical student mental health, including depression, anxiety and stress, worse than the baseline levels mentioned above. These levels, however, vary by country and region. This variance was expected by the researchers, as cultural diversity contributes profoundly to mental health. Culture impacts “ways in which health and illness are perceived, health seeking behaviors, attitudes of the consumer as well as the practitioners and mental health systems” (Gopalkrishnan, 2018). In other words, the lens through which a person interprets, copes with, and treats their mental health. Therefore, the purpose of this study was to encapsulate global data to interpret how medical students’ mental health was impacted by COVID-19. The discussion is organized similarly to how the results were outlined in order to maintain fluidity; however, it is acknowledged that some are organized by country, and other by region or continent. This form of organization is due to the quality and quantity of data found in the systematic review.

### China

Through a large meta-analysis consisting of ten cross-sectional studies involving 30,817 Chinese medical students, 29% reported depression and 21% reported anxiety; these pre-pandemic prevalence rates were similar to the findings reported globally (Zeng et al., 2019). Our review of the literature revealed a general increase in depression, anxiety and stress in this population during the COVID-19 pandemic; however, severity trended downward through the course of the pandemic. Researchers credited the initial prevalence of these mental health issues to virtual learning, increased loneliness, prolonged periods of isolation, fear of infection, poor social support, living in regions with high rates of COVID-19 exposure and infection, living alone, experiencing financial insecurities and worrying about academic delays. The protective factors against mental health issues included the availability of coping resources, online learning support, volunteer opportunities, personal resilience, and social support. A study conducted on the general population in China, during the COVID-19 pandemic, discussed the need for the following public health policies: developing and utilizing effective screening protocols at the government level to identify risk factors and provide intervention, especially targeting young individuals, individuals with lower socio-economic status, who have been quarantined, and those with severe stress (Duan et al., 2020). While this study did not assess medical students specifically, these findings could be applied at an institutional level, such as a medical school, because they take into account strategies that may be effective for Chinese individuals.

### India

Large studies on pre-pandemic rates of mental health disorders among medical students in India are scarce. One systematic review analyzed 47 studies and found elevated depression, anxiety and stress rates at 39.2, 34.5, and 51.3% respectively, with females experiencing higher rates (Sarkar et al., 2022). These prevalence rates are almost double those found globally, which may be due to cultural factors including high familial expectations, constraints for pursuing alternate interests, and the stigma surrounding mental health issues in general (Sarkar et al., 2022). Through our analysis of the literature, depression, anxiety and stress rates during the COVID-19 pandemic were variable. The majority of studies conducted during the pandemic were small-scale studies. The general trend identified was an increase in the prevalence and severity of anxiety and stress, with depression rates remaining relatively unchanged. Significant predictors of negative mental health discussed by researchers included poor sleep quality, pre-existing mental health disorders, fears of contracting and spreading COVID-19, infection with COVID-19, and direct interaction with COVID-19 patients. One systematic review that conducted data pre-pandemic emphasized regular mental health screenings with the use of standardized instruments, although did not offer solutions outside of screening (Sarkar et al., 2022).

### Rest of Asia, excluding the Middle East

Data from Pakistan, Japan, Bangladesh, Kazakhstan, Singapore, and Nepal was compiled to represent the rest of Asia, excluding the Middle East. The studies conducted in Pakistan, Kazakhstan, and Singapore revealed a decrease in student reports of depression during the COVID-19 pandemic, with researchers crediting this drop in depression rates to the transition from tradition to online learning platforms (Bolatov et al., 2021; Koh et al., 2021; Saravanan et al., 2021). This contrasts what was found in Japan, where students were more likely to be depressed and anxious if they had concerns about the switch to online learning (Nishimura et al., 2021). Rates of anxiety increased during the pandemic in Pakistan, Japan, Nepal, and Bangladesh. The researchers reported that students were at risk of worsening anxiety symptoms if they lived alone during the pandemic or worried about becoming infected with COVID-19. While it is acknowledged that Asia is a massive continent with vastly diverse cultures, researchers have made some generalized statements about Asians and mental health. For instance, researchers have investigated how emotional expression impacts mental health, citing shame as one of the reasons why Asian individuals are hesitant to seek professional help (Gopalkrishnan, 2018). Furthermore, Asian cultures may be more collective and family oriented, with a heavy focus on spirituality, impacting the way in which mental health is interpreted and treated (Gopalkrishnan, 2018).

### Middle East

Pre-pandemic levels of anxiety, depression and stress in the Middle East were variable and challenging to find. Studies in Turkey, Jordan, Saudi Arabia, Iran, and Lebanon reported high levels of anxiety, stress, and depression during the pandemic with an overall negative impact on students’ psychological state. Similar to what studies in other countries found, many of the students disliked online learning platforms and had worse rates of mental health problems if they lived alone during the pandemic. It is postulated that mental health research in Arab cultures may be lacking due to the general stigma and differences in beliefs and actions toward the treatment of anxiety and depression (Zolezzi et al., 2018).

### South America

Two studies in South America analyzed the relationship between students’ year in school and mental health effects. The majority of students found that students that experienced their first years of medical school during the pandemic reported higher levels of depression, anxiety, and stress than students in their last years of school.

### North America

Students in North America reported high levels of anxiety, depression and stress compared to global rates, with up to 84.1% of students reporting an increase in stress and anxiety and 58% of students reporting worsening depression. Risk factors included female gender, those with acquaintances diagnosed with COVID-19 and those with pre-existing mental health conditions. The increase in prevalence of anxiety, depression and stress may indicate that medical students in North America are especially susceptible to the emotional consequences of pandemics. Numerous studies found from the United States highlighted that, indeed, medical student mental health had worsened during COVID-19; however, the call for reform and policy change lacked evidence-based examples. For instance, one such study highlighted the need for “supplying students with resources, including counseling, peer advocacy, and support” (Halperin et al., 2021). In fact, most programs likely had mental health “resources” prior to the pandemic; therefore, this study is a call on medical schools, especially those that are located in countries with real-time access to mental health care, to trial interventions and identify which ones actually work. For instance, in a country like the United States, one that claims to value mental health and has worked to destigmatize mental illness, research needs to include interventions and practical applications of data. Another pandemic is inevitable and steps should be taken to minimize damage.

### Europe

Students in Ireland, Italy, and Germany were included in this review, and were found to experience moderate to severe stress, and an increase in anxiety and depressive symptoms, notably citing academic context to be a significant source of psychological distress. Future studies could analyze Eastern European medical student mental health, as these countries were underrepresented in the current literature.

### Africa and Australia

Minimal results were found from Australia and Africa. As expected, medical students in Australia experienced moderate psychological stress. One study conducted in Libya revealed that a majority of students experienced some degree of anxiety and depression, with almost 100% of students reporting depression and 22.7% reporting some form of suicidal ideation. These are the highest prevalence rates found in this systematic review. The COVID-19 pandemic occurred during the Libyan Civil War which may account for these towering numbers, as war can have drastic effects on one’s well-being. Research conducted on refugees from Africa has indicated that this group might be more reluctant to talk therapy, as there may be a perception that expressing emotions and talking about painful topics causes more harm than good (Gopalkrishnan, 2018). These researchers have also highlighted a general mistrust in clinicians as a product of perpetual racism as evidenced by historical and current persecution of African individuals (Gopalkrishnan, 2018).

### Strengths

This systematic review analyzed 47 articles from 24 different countries around the globe. The results discussed in this systematic review are consistent with prevalence rates reported in other systematic reviews on the impact of COVID-19 on medical student mental health. This study is important because it takes into account the global population. While a decline in mental health was expected, the takeaway message from this review is that culture does indeed influence mental health in many ways. It impacts attitudes and beliefs about mental health in general, the symptoms, understanding and treatment associated with distinct conditions, and one’s willingness to seek treatment. This review highlighted the differences in prevalence of anxiety and depression in the global medical student population, and shed light on gaps in the data within certain countries and regions.

### Limitations

This systematic review is limited due to the inherent bias that exists with survey studies, as well as use of many different measures of mental health without proper standardization. This review analyzed the results of self-reported mental health conditions, whereas the gold standard for establishing a diagnosis involves clinical interviewing to measure psychiatric symptoms. Many different survey formats were analyzed in this paper, which may have resulted in varying levels of mental health problems between surveys, leading to under and over representations in the literature without an objective scale being used. In addition, sample sizes and response rates varied between surveys, where the larger studies or studies with higher response rates conducted might be better representations of the sample population. The majority of the studies were conducted during or after the COVID-19 pandemic and failed to gather baseline data to compare pre-and post-pandemic levels and, generally, there was a large gap in the literature assessing medical student mental health prior to the pandemic. Finally, there are clear gaps in the existing literature with certain regions and countries. Whereas countries like China and the United States had ample research poured into this topic, South American and European data was scarce. Therefore, a true assessment of global medical student mental health cannot be made as many countries are not represented adequately.

## Conclusion and implications

Our hypothesis that medical student mental health was negatively impacted by the COVID-19 pandemic in terms of anxiety, depression and stress, was found to be correct. This systematic review is important because it highlights that medical students are particularly at risk for experiencing mental health consequences, as they experience higher prevalence rates of mental health conditions compared to the general public at baseline. These outcomes are due to a myriad of factors including the disruption of clinical training and transition to online learning, adjustment to social isolation, experiencing financial constraints, having poor social support, caring for COVID-19 patients, having challenges to accessing care, being an preclinical student, female gendered students, exposure to high-risk environments, and lacking abilities to effectively cope during unprecedented times. Protective factors included living with family during isolation periods, having ample social support, and participating in coping activities such as regular exercise. This review suggests that there should be an increased awareness and focus on medical student mental health, especially during public health emergencies such as the COVID-19 pandemic. In order to avoid student physician burn out, schools should consider providing more resources such as access to counselors and professional mental health care, as well as encourage students to employ protective coping strategies. Many of the articles highlighted the importance of utilizing validated screening protocols at the institutional level. For example, schools could start to require yearly PHQ-9 and GAD-7 screenings for their students; as titers must be routinely checked to prevent serious infection, students should also be screened for severe anxiety and depression.

As described above, mental health on a global scale generally worsened during the COVID-19 pandemic. While many researchers discussed the need for effective screening procedures to identify risk and provide intervention strategies, those intervention strategies were hardly discussed or entertained. Rather than studying prevalence data for an already at-risk population, it would be more beneficial to employ intervention strategies and monitor the effects of therapy, medication management or other accessible coping resources. Or, rather than applying these Band-Aids, so to speak, it is worth considering change at an institutional level. It would be worth determining which specific aspects of medical school during a global pandemic were the most impactful on mental health and broadly implementing them. For instance, if mental health was made worse by online learning, incorporating in-person labs or lectures back into the schedule should be considered while weighing the risk of mental illness with the risk of contracting COVID-19. Lastly, the topic of global mental health should be addressed in countries like the United States, a country with incredible cultural diversity, as culture impacts every aspect of mental health. In order for U.S. schools to take care of their global students, they must take these cultural considerations into account before inciting change.

## Data availability statement

The raw data supporting the conclusions of this article will be made available by the authors, without undue reservation.

## Author contributions

DP, MB, MZ, VB, VK, and KC contributed to data curation, formal analysis, and investigation. RR contributed to project administration and supervision. All authors contributed to conceptualization and writing—review and editing.
