# Supplementary material for: COVID-19 and mental health: A systematic review of international medical student surveys
Source: Front Psychol. 2022 Nov 25;13:1028559. doi: 10.3389/fpsyg.2022.1028559 (PMC9732539; doi:10.3389/fpsyg.2022.1028559)
Supplement: Supplementary file 1 [file Table_1.DOCX]

**Identification of studies via databases**

Records removed *before screening*:

Duplicate records removed (n = 101)

Records identified from:

Databases* (n = 832)

**Identification**

Records screened

(n = 731)

Records excluded**

(n = 273)

Reports sought for retrieval

(n = 458)

Reports not retrieved

(n = 0)

**Screening**

Reports assessed for eligibility

(n = 458)

Reports excluded***

(n = 411)

Studies included in review

(n = 47)

**Included**

*Breakdown by source: PubMed (n = 220), EMBASE (n = 354), PsycINFO (n = 13), MEDLINE Complete (n = 206), Global Health (n = 39).

**Exclusion was done manually by researchers. No automation tools were used. Reasons included (1) did not involve medical students, (2) did not study the impact of COVID-19, (3) not accessible in English, (4) publications that were not full-text (ie conference abstracts)

*** Reasons for exclusion included (1) did not involve measurements of anxiety, depression, or stress, (2) medical students were grouped with non-medical students, (3) self-reported anxiety, depression, or stress.
