# Supplementary material for: COVID-19 and mental health: A systematic review of international medical student surveys
Source: Front Psychol. 2022 Nov 25;13:1028559. doi: 10.3389/fpsyg.2022.1028559 (PMC9732539; doi:10.3389/fpsyg.2022.1028559)
Supplement: Supplementary file 3 [file Data_Sheet_1.pdf]

S1 Appendix. Study Characteristics

Title	Authors	Country	Population (School, Year in School)	Sample Method	Sample Size	Response Rate	Gender (Male: Female)	Age (Mean; standard deviation)
**The psychological impact of the COVID-19 epidemic on college students in China**	Cao et. al	China	Changzhi medical college, Undergraduates	Random	7143	100%	30.35% : 69.65%	Not reported
**COVID-19 and the mental well-being of Australian medical students: impact, concerns and coping strategies used**	Lyons et. al	Australia	University of Western Australia, all medical students	Non-random	297	37.50%	Not reported	24 (not reported)
**Online-Learning due to COVID-19 Improved Mental Health Among Medical Students**	Bolatov et. al	Kazakhstan	Astana Medical Univeristy, all medical students	Non-random	619 and 798	Not reported	25% : 75%; 24.3% : 75.7%	19.14 (not reported) and 20.37 (not reported)
**The psychological impact of the COVID-19 pandemic on medical students in Turkey**	Fuat Torun and Sebahat Dilek Torun	Turkey	Faculty of Medicine of Istanbul Yeni Yüzyıl University, all medical students	Non-random	275	63.51%	39.3% : 60.7%	22.10; 2.69
**Association of COVID-19 Pandemic with undergraduate Medical Students' Perceived Stress and Coping**	Abdulghani et. al	Saudi Arabia	King Saud University (KSU), all medical students	Non-random	243	Not reported	67.1% : 32.9%	20.6; 1.6
**Medical education in times of COVID-19: German students' expectations - A cross-sectional study**	Loda et. al	Germany	"Half of german medical institutions", all years	Non-random	372	55.10%	24.7% : 75.0%	23.92; 4.21
**Factors associated with the mental health status of medical students during the COVID-19 pandemic: a cross-sectional study in Japan**	Arima et. al	Japan	Showa University School of Medicine, all years	Non-random	571	79.86%	61.1% : 38.9%	Not Reported
**Effects of the COVID-19 pandemic on medical students: a multicenter quantitative study**	Harries et. al	USA	6 US medical schools, students in clinical years	Non-random	741	29.50%	35.1% : 63.9%	25-29; not reported
**Anxiety and Depression during COVID-19 Pandemic among Medical Students in Nepal**	Risal et. al	Nepal	7 medical schools, all years	Non-random	416	Not reported	57.7% : 42.3%	22.2; 2.1
**Psychological Impact of the Civil War and COVID-19 on Libyan Medical Students: A Cross-Sectional Study**	Elhadi et. al	Libya	15 medical schools, all years	Non-random	2430	69.42%	21.03% : 78.97%	23.30; 2.61
**Repercussions of the COVID-19 pandemic on the well-being and training of medical clerks: a pan-Canadian survey**	Abbas et. al	Canada	10 medical schools, clerks with suspended rotations	Non-random	677	Not reported	29.3% : 70.7%	20-25; not reported
**Immediate psychological responses during the initial period of the COVID-19 pandemic among Bangladeshi medical student**	Safa et. al	Bangladesh	Any Bangladeshi medical schools, all years	Random	425	Not reported	37.6% : 62.4%	22; 1.81
**Impact of COVID-19 on medical students' mental wellbeing in Jordan**	Seetan et. al	Jordan	Any medical school in Jordan, all years	Random	553	Not reported	40.1% : 59.9%	20.7; 1.9
**Levels of stress in medical students due to COVID-19**	O'Byrne et. al	Ireland	UCD school of medicine, all years	Non-random	165	26.32%	37.6% : 62.4%	22.57; 2.818

**Perception of the study situation and mental burden during the COVID-19 pandemic among undergraduate medical students with and without mentoring**	Guse et. al	Germany	University Medical Center Hamburg-Eppendorf, years 2-4	Non-random	543	45.50%	34.3% : 65.7%	21-25; not reported
**Anxiety, PTSD, and stressors in medical students during the initial peak of the COVID-19 pandemic**	Lee et. al	USA	6 US medical schools, students in clinical years	Non-random	741	29.50%	35.1% : 63.9%	25-29; not reported
**Anxiety and Gastrointestinal Symptoms Related to COVID-19 during Italian Lockdown**	Abenavoli et. al	Italy	University of Magna Graecia School of Medicine, all years	Non-random	354	Not reported	31.4% : 68.6%	23.93; 3.67
**The Effects of Coronavirus Disease 2019 Outbreak on Medical Students**	Bilgi et. al	Turkey	Bezmialem Vakif University, all years	Non-random	178	Not reported	28.7% : 71.3%	21 (median); not reported
**The Association Between Social Support, COVID-19 Exposure, and Medical Students' Mental Health**	Yin et. al	China	Any undergraduate or graduate medical students	Non-random	5982	Not reported	40.0% : 60.0%	22; 2.5
**Assessing the Psychological Impacts of COVID-19 in Undergraduate Medical Students**	Guo et. al	USA	24 medical schools, all years	Random	929	Not reported	Not reported	Not reported
**Medical student wellness in the United States during the COVID-19 pandemic: a nationwide survey**	Nikolis et. al	USA	112 medical schools, all years	Random	1377	Not reported	33% : 67%	Not reported
**COVID-19 pandemic and its aftermath: Knowledge, attitude, behavior, and mental health-care needs of medical undergraduates**	Shailaja et. al	India	Bengaluru medical school, all years	Non-random	530	81.60%	42.6% : 57.4%	20.57; 1.85
**The educational and psychological impact of the COVID-19 pandemic on medical students: A descriptive survey at the American University of Beirut**	Bachir et. al	Lebanon	AUBFM, all years	Non-random	168	37.50%	46.5% : 53.5%	Not reported (93.5% between 20-24)
**The effect of COVID-19 on medical students' education and wellbeing: a cross-sectional survey**	ElHawary et. al	Canada	13 Canadian medical schools, all years	Non-random	248	Not reported	37% : 63%	24.5; 3.5
**Prevalence of Anxiety and Depression Among Medical Students During the Covid-19 Pandemic: A Cross-Sectional Study**	Halperin et. al	USA	40 US medical schools, all years	Non-random	1428	9.30%	33.3% : 66.7%	22.3; 9
**Mental health in medical students during COVID-19 quarantine: a comprehensive analysis across year-classes**	Perissotto et. al	Brazil	Jundiai Medical School, all years	Non-random	347	51.00%	34.1% : 65.9%	22.6; 2.7
**Predictive Factors for Impaired Mental Health among Medical Students during the Early Stage of the COVID-19 Pandemic in Morocco**	Essangri et. al	Morocco	7 medical schools, all years	Non-random	549	4.05%	26% : 74%	22; 3
**Association between perceived stress and depression among medical students during the outbreak of COVID-19: The mediating role of insomnia**	Liu, et al.	China	Medical students from three Chinese medical universities	Non-random	29663	Not reported	10185 M : 19478 F	21.46; 2.50
**Perceived Stress Among Chinese Medical Students Engaging in Online Learning in Light of COVID-19**	Wang, et al.	China	Medical students from 3 Chinese medical schools	Non-random	369	48.80%	150 M : 219 F	20.2; 1.41

**Attitudes towards COVID-19 precautionary measures and willingness to work during an outbreak among medical students in Singapore: a mixed-methods study**	Koh, et al.	Singapore	Medical students at a Singaporean medical school	Non-random	263	Not reported	123 M : 140 F	21.9, SD not reported
**Depressive Symptoms, Sleep Quality and Diet During the 2019 Novel Coronavirus Epidemic in China: A Survey of Medical Students[50]**	Xie, et al.	China	Medical students at Kunming University	Non-random	1026	Not reported	373 M : 653 F	Not reported
**Impact of COVID-19 pandemic on happiness and stress: comparison of preclinical and clinical medical students[51]**	Isaradisaikul, et al.	Thailand	Medical student at Chiang Mai University	Non-random	369	25.50%	103 M : 128 F	19, SD not reported
**Psychological Burden and Experiences Following Exposure to COVID-19: A Qualitative and Quantitative Study of Chinese Medical Student Volunteers**	Zhang, et al.	China	Medical students at a Chinese medical college	Non-random	1041	Not reported	496 M : 545 F	21.34; 2.0
**Impact of the Perceived Mental Stress During the COVID-19 Pandemic on Medical Students' Loneliness Feelings and Future Career Choice: A Preliminary Survey Study**	Zheng, et al.	China	Medical students from 12 Chinese medical schools (Study 1), Fuijan Medical University (Study 2)	Non-random	906 (Study 1), 354 (Study 2)	Not reported	312 M : 594 F (Study 1), 196 M : 158 F (Study 2)	21.75; 1.90 (Study 1), 21.09; 1.63 (Study 2)
**Factors associated with mental health in Peruvian medical students during the COVID-19 pandemic: a multicentre quantitative study**	Huarcaya-Victoria, et al.	Peru	Medical students from 8 Peruvian medical schools	Non-random	1549	79.90%	390 M : 848 F	21.4; 3.7
**A longitudinal study on psychological burden of medical students during COVID-19 outbreak and remission period in China**	Zhang, et al.	China	Medical students at Shantou University Medical College	Non-random	1069 (OP), 1511 (RP)	94.85% (OP), 94.85% (RP)	442 M : 627 F (OP), 710 M : 801 F (RP)	- OP: 20.93; 1.64<br>- RP: 21.21; 1.69
**The deep impact of the COVID-19 pandemic on medical students: An online cross-sectional study evaluating Turkish students' anxiety[52]**	Tuncel, et al.	Turkey	Medical students from 70 different universities in Turkey	Non-random	3105	Not reported	1343 M : 1762 F	22.37; 2.46
**Coping Styles for Mediating the Effect of Resilience on Depression Among Medical Students in Web-Based Classes During the COVID-19 Pandemic: Cross-sectional Questionnaire Study**	Zhao, et al.	China	Medical students at China Medical University	Non-random	666	92.50%	262 M : 404 F	20, SD not reported
**Impact of the COVID-19 Pandemic on the Psychological Distress of Medical Students in Japan: Cross-sectional Survey Study**	Nishimura, et al.	Japan	Medical students at Okayama University SOM	Non-random	473	66%	161 F : 311 M	22.0; 3.3
**Emergency remote learning in anatomy during the COVID-19 pandemic: A study evaluating academic factors contributing to anxiety among first year medical students[53]**	Srivastava et. al	India	First year medical students at Christian Medical College	Non-random	97	Not reported	46 M : 51 F	19.15, SD not reported
**Depression and anxiety among students community during COVID-19 pandemic lockdown in Tamil nadu- A web based descriptive cross sectional study**	Saravanan, et al.	India	Medical students in and around Chennai city	Non-random	500 students	Not reported	44% : 56%	Not reported
**The Influence of Covid-19 Lockdown on Body Mass Index, Depression, Anxiety and Stress among Medical Students.**	Masud, et al	Pakistan	Medical students at CMH Kharian Medical College	Non-random	233 students	Not reported	Not reported	Not reported

**Medical students' awareness of COVID-19 against the background of remote learning[54]**	Kuchma, et al	Russia	Medical students at Sechenov First Moscow State Medical University	Non-random	142	Not reported	65M : 77F	Not reported
**P.700 Prevalence of depression in medical students during lockdown in Brazil due to COVID-19 pandemic**	Miskulin, et al	Brazil	Medical students at Jundiai Medical School	Non-random	347	51%	Not reported	Not reported
**The Psychological Impact of the Covid-19 Lockdown on Medical Students of a College in North India**	Kumar, et al	India	Medical students at Medical College of North India	Non-random	331	73.60%	130M : 201F	Not reported
**A survey on anxiety and depression level among South Indian medical students during the COVID 19 pandemic[55]**	Nisha, et al	India	South Indian medical students	Non-random	359	Not reported	181M : 178F	Not reported
**Study of depression, anxiety and stress among first year medical students in Government Medical College, Himachal Pradesh during COVID-19 pandemic.[56]**	Rana, et al	India	1st year medical students at Government Medical College, Himachal Pradesh	Non-random	110	Not reported	49M : 61F	19.45; 1.55
