# Supplementary material for: COVID-19 and mental health: A systematic review of international medical student surveys
Source: Front Psychol. 2022 Nov 25;13:1028559. doi: 10.3389/fpsyg.2022.1028559 (PMC9732539; doi:10.3389/fpsyg.2022.1028559)
Supplement: Supplementary file 4 [file Data_Sheet_2.PDF]

S2 Appendix. Prevalence Data and Relevant Results

Title	Authors	Sample size	Instruments use	Prevalence: Stress	Instruments use	Prevalence: Anxiety	Instruments use	Prevalence: Depression	Related Factors Assessed	Outcomes and other results
**The psychological impact of the COVID-19 epidemic on college students in China**	Cao et. al	7143	Not assessed	Not assessed	GAD-7	Mild: 21.3%, moderate: 2.7%, severe: 0.9%	Not assessed	Not assessed	Place of residence, steady family income, living with parents, relative or acquaintance infected with COVID-19, economics, academic delays, influence on daily life, social support	24.9% experienced anxiety
**COVID-19 and the mental well-being of Australian medical students: impact, concerns and coping strategies used**	Lyons et. al	297	K10	mean score: 20.6 (moderate psychological distress); Low (26%), moderate (37%), high (26%), very high (11%)	Not assessed	Not assessed	Not assessed	Not assessed	Impact on different areas of life, concerns related to COVID-19, coping strategies	1st year medical students had highest psychological distress scores (mean: 23.1 = moderate)
**Online-Learning due to COVID-19 Improved Mental Health Among Medical Students**	Bolatov et. al	619 and 798	Not assessed	Not assessed	GAD-7	Traditional (42.3%): mild (30.5%), moderate (15.4%), severe (17.6%). Online (15.5%): mild (27.1%), moderate (9.9%), severe (5.6%)	PHQ-9	Traditional (49.3%): mild (31%), moderate (23.4%), moderately severe (13.2%), severe (12.7%). Online (27.6%): mild (31.9%), moderate (16.4%), moderately severe (7.2%), severe (4.0%)	CBI-S (burnout), PHQ-15, Fear of COVID-19	Lower rates of depression and anxiety during online learning. Burnout: personal burnout (55.7%), studies related burnout (54.9%), colleague-related burnout (13.1%), teacher related burnout (24.1%)
**The psychological impact of the COVID-19 pandemic on medical students in Turkey**	Fuat Torun and Sebahat Dilek Torun	275	PSS	Prevelance not reported.	Not assessed	Not assessed	Not assessed	Not assessed	IES-R	Mean scores of women's total PSS and IES-R were higher than men
**Association of COVID-19 Pandemic with undergraduate Medical Students' Perceived Stress and Coping**	Abdulghani et. al	243	K10	no stress 109 (44.9%), mild stress 75 (30.9%), moderate stress 28 (11.5%), and severe stress 31 (12.8%)	Not assessed	Not assessed	Not assessed	Not assessed	Coping strategies, academic variables compared to stress levels	Female stress level much higher (40%) as compared to the male students (16.6%)
**Medical education in times of COVID-19: German students' expectations - A cross-sectional study**	Loda et. al	372	Not assessed	Not assessed	STAI, GAD7	STAI: mean = 45.12 (high), SD = 4.73	Not assessed	Not assessed	Internal external locus of control scale (likert scale 0 to 5)	High (> 7) internal level of control (mean = 8.19) normal external level of control (mean = 4.31)
**Factors associated with the mental health status of medical students during the COVID-19 pandemic: a cross-sectional study in Japan**	Arima et. al	571	Not assessed	Not assessed	Not assessed	Not assessed	Not assessed	Not assessed	K-6 (psychological distress), Rosenberg Self-Esteem Scale, General Self-Efficacy Scale	28.5% scored greater than 5 (significant distress). Higher RSES correlated lower K-6. Higher GSES correlated higher K-6
**Effects of the COVID-19 pandemic on medical students: a multicenter quantitative study**	Harries et. al	741	Not assessed	Not assessed	Self reported	Atleast "somewhat anxious" (84.1%)	Not assessed	Not assessed	Education disruption	74.7% reported significant disruption
**Anxiety and Depression during COVID-19 Pandemic among Medical Students in Nepal**	Risal et. al	416	Not assessed	Not assessed	HADS-Anxiety	Overall prevelance 11.8%	HADS-Depression	Overall prevalence 5.5%	HADS caseness	26.7% had either anxiety, depression, or anxiety with comorbid depression
**Psychological Impact of the Civil War and COVID-19 on Libyan Medical Students: A Cross-Sectional Study**	Elhadi et. al	2430	Not assessed	Not assessed	GAD-7	Overall prevelance 64.5%; mild 37.5%; moderate 16%; severe 11%	PHQ-9	Overall prevalence 78%; mild 35.2%; moderate 21.2%; moderately severe 11.9%; severe 9.7%.	Psychological stress due to the civil war	None
**Repercussions of the COVID-19 pandemic on the well-being and training of medical clerks: a pan-Canadian survey**	Abbas et. al	677	Not assessed	Not assessed	Not assessed	Not assessed	Not assessed	Not assessed	WHO well-being index, stress management and resources, stressors	Approximately 45% reported higher stress levels than pre-pandemic
**Immediate psychological responses during the initial period of the COVID-19 pandemic among Bangladeshi medical student**	Safa et. al	425	Not assessed	Not assessed	HADS	Overall prevelance 65.9%, mild 27.3%, moderate 26.8%, severe 11.8%	HADS	Overall prevalence 49.9%, severe 3.3%	None	None
**Impact of COVID-19 on medical students' mental wellbeing in Jordan**	Seetan et. al	553	K10	Overall prevalence 86.6%, mild 16.5%, moderate 20.1%, severe 50.3%	Not assessed	Not assessed	Not assessed	Not assessed	None	None
**Levels of stress in medical students due to COVID-19**	O'Byrne et. al	165	Self reporting	Overall prevalence 90.9%, mild 36.4%, moderate 31.5%, severe 23%	Not assessed	Not assessed	Not assessed	Not assessed	Coping mechanisms	Engaged with positive, negative, or both positive and negative coping mechanisms (n=67, n=15, n=83)
**Perception of the study situation and mental burden during the COVID-19 pandemic among undergraduate medical students with and without mentoring**	Guse et. al	543	Not assessed	Not assessed	Not assessed	Not assessed	Not assessed	Not assessed	PHQ-4	Anxiety and depression symptoms: prevalence 44%, mild 33.9%, moderate 7.4%, severe 2.8%.
**Anxiety, PTSD, and stressors in medical students during the initial peak of the COVID-19 pandemic**	Lee et. al	741	Self reporting	Overall prevalence 84.1%.	GAD-7	Overall prevalence 60.4%, mild 34.4%, moderate 16.1%, severe 9.5%	Not assessed	Not assessed	PC-PTSD (PTSD risk)	25.4% positive for PTSD screen

**Anxiety and Gastrointestinal Symptoms Related to COVID-19 during Italian Lockdown**	Abenavoli et. al	354	Not assessed	Not assessed	SHAI	Overall prevalence 48.9%; score mean 18.12 (SD = 7.290, anxiety state ≥ 18)	Not assessed	Not assessed	None	None
**The Effects of Coronavirus Disease 2019 Outbreak on Medical Students**	Bilgi et. al	178	Not assessed	Not assessed	GAD-7	Overall prevalence 74.2%, mild 37.1%, moderate 17.4%, severe 19.7%	PHQ-9	Overall prevalence 58.4%, mild 23.0%, moderate 21.9%, severe 13.5%	None	None
**The Association Between Social Support, COVID-19 Exposure, and Medical Students' Mental Health**	Yin et. al	5982	Not assessed	Not assessed	GAD-7	Overall prevalence 22.8%, mild 18.6%, moderate 2.3%, severe 1.8%	PHQ-9	Overall prevalence 35.1%, mild 25.3%, moderate 6.6%, severe 3.2%	SSRS (social support rating scale)	Increased social support was associated with decreased severity of depression and anxiety symptoms
**Assessing the Psychological Impacts of COVID-19 in Undergraduate Medical Students**	Guo et. al	929	PSS-4	Mean score was 7.25 /16 (SD = 3.05)	GAD-7	Overall prevalance 66.08%, mild 34.98%, moderate 19.25%, severe 11.85%	Not assessed	Not assessed	Stress association with class	First year students had statistically lower levels of stress than that of second, third, and fourth year students (p < 0.05)
**Medical student wellness in the United States during the COVID-19 pandemic: a nationwide survey**	Nikolis et. al	1377	Not assessed	Not assessed	Not assessed	Not assessed	Not assessed	Not assessed	Custom survey: "Medical Student Wellness During the COVID-19 Pandemic"	Average pre-pandemic wellness score was 6.95/10 (SD = 1.53), average pandemic wellness score was 5.87/10 (SD = 2.01)
**COVID-19 pandemic and its aftermath: Knowledge, attitude, behavior, and mental health-care needs of medical undergraduates**	Shailaga et. al	530	DASS	Overall prevalence 13.0%, mild 4.7%, moderate 4.0%, severe 3.2%, extremely severe 1.1%	DASS	Overall prevalence 20.7%, mild 5.8%, moderate 9.1%, severe 2.6%, extremely severe 3.2%	DASS	Overall prevalence 23.2%, mild 7.5%, moderate 7.4%, severe 3.8%, extremely severe 4.5%	Quality of life: EUROHIS-QOL-8 scale	3.9 (SD 0.6)
**The educational and psychological impact of the COVID-19 pandemic on medical students: A descriptive survey at the American University of Beirut**	Bachir et. al	168	Custom survey	Majority reported more stress in academic and social life. Year 3 reported nervousness with hospital rotations.	Not assessed	Not assessed	Not assessed	Not assessed	None	None
**The effect of COVID-19 on medical students' education and wellbeing: a cross-sectional survey**	ElHawary et. al	248	Self reported	69% with psychiatric history; 41% with no psychiatric history	Self reported	69% with psychiatric history; 41% with no psychiatric history	Self reported	66% with psychiatric history; 42% with no psychiatric history	None	None
**Prevalence of Anxiety and Depression Among Medical Students During the Covid-19 Pandemic: A Cross-Sectional Study**	Halperin et. al	1428	Not assessed	Not assessed	GAD-7	Overall prevalence 30.6%	PHQ-9	Overall prevalence 24.3%	None	None
**Mental health in medical students during COVID-19 quarantine: a comprehensive analysis across year-classes**	Perissotto et. al	347	Not assessed	Not assessed	HADS-Anxiety	Overall prevalence 59.7%	HADS-Depression	Overall prevalence 36.0%	None	None
**Predictive Factors for Impaired Mental Health among Medical Students during the Early Stage of the COVID-19 Pandemic in Morocco**	Essangri et. al	549	K6	Overall prevalence 69%, moderate 47.5%, serious 21.5%	GAD-7	Overall prevalence 62.3%, mild 36.6%, moderate 15.8%, severe 9.8%,	PHQ-9	Overall prevalence 74.7%, mild 29%, moderate 21.5%, severe 24.2%	None	None
**Association between perceived stress and depression among medical students during the outbreak of COVID-19: The mediating role of insomnia**	Liu, et al.	29663	Perceived Stress Scale 14 (PSS-14)	Not reported	Not assessed	Not assessed	PHQ-9	Not reported	Perceived stress and depression correlation with each other and insomnia	- Depression was positively correlated with perceived stress (r=0. 512, P < 0.001) and insomnia (r=0.679, P < 0.001)<br>- Perceived stress was positively correlated with insomnia (r=0. 399, P < 0.001).
**Perceived Stress Among Chinese Medical Students Engaging in Online Learning in Light of COVID-19**	Wang, et al.	369	Perceived Stress Scale (PSS-10)	17.39 (moderate to high stress)	Not assessed	Not assessed	Not assessed	Not assessed	Associations between perceived stress and demographics, stressors, online learning, attitude	- Gender (female, β= 0.156, P < 0.001) and grade (junior students, β= 0.108, P= 0.007) were positive predictors of PS.<br>- The persistence, attitude and flexibility of students' online learning played a negative role in PS.<br>- Psychosocial stressors (β= 0.212, P= 0.003) and health-related stressors (β= 0.166, P= 0.008) increased students' PS.<br>- For the online learning environment, teaching presence (β= −0. 140, P= 0.025) and social presence (β= −0.160, P= 0.033) were significant and negative predictors correlated with PS.<br>- Cognitive presence (β= 0.259, P= 0.005) positively predicted PS
**Attitudes towards COVID-19 precautionary measures and willingness to work during an outbreak among medical students in Singapore: a mixed-methods study**	Koh, et al.	263	Not assessed	Not assessed	Modified GAD-7 (added "due to COVID" at end of each question)	Not reported	Not assessed	Not assessed	Anxiety-related to COVID-19 associations with perceived information adequacy	- Students with perceived information adequacy reported a lower score regarding COVID-19 anxiety (t = − 2.90, p < 0.001)
**Depressive Symptoms, Sleep Quality and Diet During the 2019 Novel Coronavirus Epidemic in China: A Survey of Medical Students**	Xie, et al.	1026	Not assessed	Not assessed	Not assessed	Not assessed	Chinese version of Self-Rated Depression scale	22.4% (depressive symptoms)	Depressive symptom association with gender, age, location, COVID-19 related factors, poor sleep quality, poor diet	- Gender (OR = 1.45, 95% CI: 1.07–1.96) and degree of focus on COVID-19 (OR = 1.47, 95% CI: 1.08–2.00) were significantly associated with depressive symptoms
**Impact of COVID-19 pandemic on happiness and stress: comparison of preclinical and clinical medical students**	Isaradisaikul, et al.	369	Thai Stress Questionnaire (Thai-ST5)	4 (low stress)	Not assessed	Not assessed	Not assessed	Not assessed	None	None

**Psychological Burden and Experiences Following Exposure to COVID-19: A Qualitative and Quantitative Study of Chinese Medical Student Volunteers**	Zhang, et al.	1041	DASS-21	11.1% (mild to extremely severe)	DASS-21	20.2% (mild to extremely severe)	DASS-21	26.8% (mild to extremely severe)	None	None
**Impact of the Perceived Mental Stress During the COVID-19 Pandemic on Medical Students' Loneliness Feelings and Future Career Choice: A Preliminary Survey Study**	Zheng, et al.	906 (Study 1), 354 (Study 2)	14-item Chinese Perceived Stress Scale (CPSS)	Not assessed	Not assessed	Not assessed	Not assessed	Not assessed	Association of mental stress with perceived influence on career choice, loneliness, COVID-19	Study 1:<br>- Significant positive relations between the scores on perceived mental stress (M = 38.66, SD = 6.55) and loneliness feelings (M = 40.66, SD = 9.10; r = 0.62, p < 0.001)<br>- Significant positive correlations between the score of perceived influence on career choice (M = 2.14, SD = 1.06) and perceived mental stress (r = 0.11, p = 0.001)<br>Study 2:<br>- Self-reported possibility of infection (M = 2.06, SD = 0.77) positively correlated with respondents perceived mental stress (Cronbach α = 0.857; M = 38.18, SD = 7.21; r = 0.12, p = 0.023)
**Factors associated with mental health in Peruvian medical students during the COVID-19 pandemic: a multicentre quantitative study**	Huarcaya-Victoria, et al.	1549	Not assessed	Not assessed	GAD-7	528 (43%) of the students reported no anxiety symptoms, 475 (38%) had mild symptoms, 177 (14%) moderate symptoms, and 58 (5%) severe symptoms	PHQ-9	327 (26%) of the students reported no depressive symptoms, 490 (40%) had mild symptoms, 242 (20%) moderate, 137 (11%) moderately severe, and 42 (3%) severe.	Anxiety and depression association with age, gender, year in school, religion, living status, feeling fear about various fators	- Women were a factor associated with clinically relevant symptoms of depression (aOR = 1.34; 95%CI, 1.01-1.75)<br>- Being in pre-clinical years (1st-3rd year) were a factor associated with clinically relevant symptoms of depression (aOR = 2.50; 95%CI, 1.38-4.52), anxiety (aOR = 2.35; 95%CI, 1.71-3.22)<br>- Not having family economic stability was associated with clinically relevant symptoms of depression (aOR = 1.59; 95%CI, 1.19-2.11), anxiety (aOR = 2.05; 95%CI, 1.47-2.86)<br>- The fear of damaging the medical training since the pandemic was associated with clinically relevant symptoms of depression (aOR = 2.67; 95%CI, 1.16-2.19), anxiety (aOR = 3.12; 95%CI, 2.01-4.76)
**A longitudinal study on psychological burden of medical students during COVID-19 outbreak and remission period in China**	Zhang, et al.	1069 (OP), 1511 (RP)	DASS-21	- OP: Mean score of 1.45±2.88 (no presence of stress)<br>- RP: Mean score of 2.89±3.80 (no presence of stress)	DASS-21	- OP: Mean score of 0.95 ±2.27 (no presence of anxiety)<br>- RP: Mean score of 1.89 ±3.30 (no presence of anxiety)	DASS-21	- RP: Mean score of 2.73±3.82 (no presence of depression)	- Increase of measures between OP and RP<br>- Factors that impacted anxiety, stress, and depression	- Stress (t=–10.94,P<0.001) and anxiety (t=–8.56,P<0.001) increased between OP and RP<br>- Common impact factors of the depression, anxiety, and stress included frequency of outdoor activities, mask-wearing adherence, self-perceived unhealthy status, and exposure to COVID-19.<br>- A diagnosis of infection was found to be a unique risk factor for anxiety
**The deep impact of the COVID-19 pandemic on medical students: An online cross-sectional study evaluating Turkish students' anxiety**	Tuncel, et al.	3105	Not assessed	Not assessed	Beck Anxiety Inventory (BAI)	1542 (49.7%) students had minimal anxiety, 844 (27.2%) had mild anxiety, 466 (15%) had moderate anxiety and 253 (8.1%) had severe anxiety.	Not assessed	Not assessed	BAI level association with year in school, knowledge of preventive measures, COVID-19 related factors	- Prevalence of anxiety was lower amongst those who reported that they were not exposed to a COVID-19-diagnosed patient (P < .0001, χ 2 = 12.623)<br>- Students who thought they did not have a higher risk for the transmission of coronavirus because they were medical students (P < .0001, χ 2 = 23.519), were less likely to be anxious
**Coping Styles for Mediating the Effect of Resilience on Depression Among Medical Students in Web-Based Classes During the COVID-19 Pandemic: Cross-sectional Questionnaire Study**	Zhao, et al.	666	Not assessed	Not assessed	Not assessed	Not assessed	PHQ-9	9.6% moderate or more severe	PHQ-9 level association with gender, adapting to web-based classes, resilience, and coping styles	- The depression scores of the male students were significantly higher than those of the female students (P=.045).<br>- The depression scores among the students who were not adapting to web-based classes were significantly higher than those among the students who were adapting to web-based classes (P<.001)<br>- Depression among medical students was significantly and negatively associated with both resilience (P<.001) and positive coping styles (P<.001), while depression was significantly and positively associated with negative coping styles among medical students (P<.001).<br>- Grade (P=.013), resilience (P=.04), positive coping styles (P<.001), and negative coping styles (P<.001) were significant predictors for depression.
**Impact of the COVID-19 Pandemic on the Psychological Distress of Medical Students in Japan: Cross-sectional Survey Study**	Nishimura, et al.	473	Not assessed	Not assessed	GAD-7	7.2% moderate or more severe	PHQ-9	15.9% moderate or more severe	Odds of being depressed/anxious and:<br>- Having concerns about a shift toward online education<br>- Saying they would request food aid from the university in the event of a COVID-19 resurgence<br>- Saying they would request mental health care resources from the university in the event of a COVID-19 resurgence	The odds of being depressed were significantly higher in those who had concerns about a shift toward online education (odds ratio [OR] 1.97, 95% CI 1.19-3.28) and in those who said they would request food aid (OR 1.99, 95% CI 1.16-3.44) and mental health care resources (OR 3.56, 95% CI 2.07-6.15) from the university in the event of the resurgence of COVID-19. Regarding generalized anxiety, the odds were higher in respondents who said they would request food aid (OR 2.50, 95% CI 1.21-5.20) and mental health care resources (OR 3.16, 95% CI 1.51-6.59).
**Emergency remote learning in anatomy during the COVID-19 pandemic: A study evaluating academic factors contributing to anxiety among first year medical students**	Srivastava et. al	97	Not assessed	Not assessed	GAD-7	43.30% had minimal, 31.96% mild, 10.31% moderate and 14.43% of students had severe anxiety	Not assessed	Not assessed	GAD-7 level association with academic interaction, learning styles (VARK) and stress-reliving factors	- Correlation between lower GAD-7 scores and enjoyment of small group discussions
**Depression and anxiety among students community during COVID-19 pandemic lockdown in Tamil nadu- A web based descriptive cross sectional study**	Saravanan, et al.	500	Not reported	Not reported	DASS21	16%	DASS21	18.20%	Anxiety and depression association with gender	Association statistical significances were not reported
**The Influence of Covid-19 Lockdown on Body Mass Index, Depression, Anxiety and Stress among Medical Students.**	Masud, et al	233	DASS-21	- Pre-COVID-19 lockdown: 4.74 (Normal stress)<br>- Post-COVID-19 lockdown: 15.59 (Severe stress)	DASS-21	- Pre-COVID-19 lockdown: 5.04 (Normal anxiety)<br>- Post-COVID-19 lockdown: 14.39 (Moderate anxiety)	DASS-21	- Pre-COVID-19 lockdown: 24.52 (Severe depression)<br>- Post-COVID-19 lockdown: 12.91 (Mild depression)	Association with age, pre/post-COVID lockdown	Statistically significant difference in depression among all age groups (17-19, 20-21, 22-24) pre/post-COVID lockown

**Medical students' awareness of COVID-19 against the background of remote learning**	Kuchma, et al	142	Not assessed	Not assessed	Self-designed questionnaire	Not reported	Not assessed	Not assessed	Various COVID-19 related factors	None
**P.700 Prevalence of depression in medical students during lockdown in Brazil due to COVID-19 pandemic**	Miskulin, et al	347	Not assessed	Not assessed	Not assessed	Not assessed	HADS	36% above cut-off for clinically relevant depressive symptoms	Association with year, age, internship scores, gender, being with family	Clinically relevant depressive symptoms (HADS >8) were:<br>- Inversely correlated with year of class, with first-years having the highest average total scores<br>- Correlated with gender (women had higher prevalence)<br>- Not decreased by being with family
**The Psychological Impact of the Covid-19 Lockdown on Medical Students of a College in North INdia**	Kumar, et al	331	IES-R (Assesses subjective distress caused by traumatic events)	- Mild psychological impact: 16.3%<br>- Moderate psychological impact: 6.1%<br>- Severe psychological impact: 16.6%	Not assessed	Not assessed	Not assessed	Not assessed	Score association with gender and various stressors associated with COVID-19	- Scores above the cutoff for mild psychological impact were associated with the following stressors: Lockdown will delay completion of degree, feeling stressed during lockdown, finding parents worried about their earnings during or after lockdown<br>- Normal scores (below the cutoff) were associated with getting to know family better during lockdown
**A survey on anxiety and depression level among South Indian medical students during the COVID 19 pandemic**	Nisha, et al	359	Not assessed	Not assessed	GAD-7	75.5% showing mild to severe anxiety symptoms	CES-D	74.6% showing mild to severe depressive symptoms	None	None
**Study of depression, anxiety and stress among first year medical students in Government Medical College, Himachal Pradesh during COVID-19 pandemic.**	Rana, et al	110	DASS-21	32% mild and above stress	DASS-21	74% mild and above anxiety	DASS-21	58% mild and above depression	Association with gender, between measures	- Higher total DASS score in females<br>- Strong correlation between scores of depression, anxiety and stress
