# Supplementary material for: COVID-19 and mental health: A systematic review of international medical student surveys
Source: Front Psychol. 2022 Nov 25;13:1028559. doi: 10.3389/fpsyg.2022.1028559 (PMC9732539; doi:10.3389/fpsyg.2022.1028559)
Supplement: Supplementary file 5 [file Data_Sheet_3.pdf]

S3 Appendix. Quality Assessment

Study	Authors	Representativeness of sample	Adequacy of response rate	Missing data within completed questionnaires	Conduct of pilot testing	Establish validity of the survey instrument
**The psychological impact of the COVID-19 epidemic on college students in China**	Cao et. al	2	1	1	4	1
**COVID-19 and the mental well-being of Australian medical students: impact, concerns and coping strategies used**	Lyons et. al	2	2	1	4	2
**Online-Learning due to COVID-19 Improved Mental Health Among Medical Students**	Bolatov et. al	1	1	1	4	1
**The psychological impact of the COVID-19 pandemic on medical students in Turkey**	Fuat Torun and Sebahat Dilek Torun	1	2	1	4	1
**Association of COVID-19 Pandemic with undergraduate Medical Students' Perceived Stress and Coping**	Abdulghani et. al	1	1	1	4	1
**Medical education in times of COVID-19: German students' expectations - A cross-sectional study**	Loda et. al	2	2	1	4	1
**Factors associated with the mental health status of medical students during the COVID-19 pandemic: a cross-sectional study in Japan**	Arima et. al	1	1	1	4	1
**Effects of the COVID-19 pandemic on medical students: a multicenter quantitative study**	Harries et. al	2	2	1	1	2
**Anxiety and Depression during COVID-19 Pandemic among Medical Students in Nepal**	Risal et. al	1	1	1	4	1
**Psychological Impact of the Civil War and COVID-19 on Libyan Medical Students: A Cross-Sectional Study**	Elhadi et. al	1	1	2	4	1
**Repercussions of the COVID-19 pandemic on the well-being and training of medical clerks: a pan-Canadian survey**	Abbas et. al	1	2	2	4	2
**Immediate psychological responses during the initial period of the COVID-19 pandemic among Bangladeshi medical student**	Safa et. al	1	1	1	4	1
**Impact of COVID-19 on medical students' mental wellbeing in Jordan**	Seetan et. al	2	1	2	4	2
**Levels of stress in medical students due to COVID-19**	O'Byrne et. al	2	3	1	1	1

**Perception of the study situation and mental burden during the COVID-19 pandemic among undergraduate medical students with and without mentoring**	Guse et. al	2	2	2	4	2
**Anxiety, PTSD, and stressors in medical students during the initial peak of the COVID-19 pandemic**	Lee et. al	2	2	2	2	1
**Anxiety and Gastrointestinal Symptoms Related to COVID-19 during Italian Lockdown**	Abenavoli et. al	1	1	2	3	3
**The Effects of Coronavirus Disease 2019 Outbreak on Medical Students**	Bilgi et. al	1	2	1	4	1
**The Association Between Social Support, COVID-19 Exposure, and Medical Students' Mental Health**	Yin et. al	2	1	1	4	1
**Assessing the Psychological Impacts of COVID-19 in Undergraduate Medical Students**	Guo et. al	1	1	1	4	1
**Medical student wellness in the United States during the COVID-19 pandemic: a nationwide survey**	Nikolis et. al	1	2	2	4	2
**COVID-19 pandemic and its aftermath: Knowledge, attitude, behavior, and mental health-care needs of medical undergraduates**	Shailaga et. al	1	2	1	4	1
**The educational and psychological impact of the COVID-19 pandemic on medical students: A descriptive survey at the American University of Beirut**	Bachir et. al	1	1	1	4	1
**The effect of COVID-19 on medical students' education and wellbeing: a cross-sectional survey**	ElHawary et. al	1	2	2	4	1
**Prevalence of Anxiety and Depression Among Medical Students During the Covid-19 Pandemic: A Cross-Sectional Study**	Halperin et. al	1	1	1	4	1
**Mental health in medical students during COVID-19 quarantine: a comprehensive analysis across year-classes**	Perissotto et. al	2	2	2	4	2
**Predictive Factors for Impaired Mental Health among Medical Students during the Early Stage of the COVID-19 Pandemic in Morocco**	Essangri et. al	1	1	2	4	2
**Association between perceived stress and depression among medical students during the outbreak of COVID-19: The mediating role of insomnia**	Liu, et al.	1	1	1	4	1

**Perceived Stress Among Chinese Medical Students Engaging in Online Learning in Light of COVID-19**	Wang, et al.	2	2	2	4	2
**Attitudes towards COVID-19 precautionary measures and willingness to work during an outbreak among medical students in Singapore: a mixed-methods study**	Koh, et al.	2	2	2	4	1
**Depressive Symptoms, Sleep Quality and Diet During the 2019 Novel Coronavirus Epidemic in China: A Survey of Medical Students**	Xie, et al.	1	1	2	4	2
**Impact of COVID-19 pandemic on happiness and stress: comparison of preclinical and clinical medical students**	Isaradisaikul, et al.	2	2	1	4	1
**Psychological Burden and Experiences Following Exposure to COVID-19: A Qualitative and Quantitative Study of Chinese Medical Student Volunteers**	Zhang, et al.	1	1	1	4	1
**Impact of the Perceived Mental Stress During the COVID-19 Pandemic on Medical Students' Loneliness Feelings and Future Career Choice: A Preliminary Survey Study**	Zheng, et al.	1	1	1	4	1
**Factors associated with mental health in Peruvian medical students during the COVID-19 pandemic: a multicentre quantitative study**	Huarcaya-Victoria, et al.	1	1	2	4	1
**A longitudinal study on psychological burden of medical students during COVID-19 outbreak and remission period in China**	Zhang, et al.	1	1	3	4	1
**The deep impact of the COVID-19 pandemic on medical students: An online cross-sectional study evaluating Turkish students' anxiety**	Tuncel, et al.	1	4	4	4	3
**Coping Styles for Mediating the Effect of Resilience on Depression Among Medical Students in Web-Based Classes During the COVID-19 Pandemic: Cross-sectional Questionnaire Study**	Zhao, et al.	1	1	4	4	1
**Impact of the COVID-19 Pandemic on the Psychological Distress of Medical Students in Japan: Cross-sectional Survey Study**	Nishimura, et al.	1	4	2	1	1
**Emergency remote learning in anatomy during the COVID-19 pandemic: A study evaluating academic factors contributing to anxiety among first year medical students**	Srivastava et. al	1	1	4	4	4

**Depression and anxiety among students community during COVID-19 pandemic lockdown in Tamil nadu- A web based descriptive cross sectional study**	Saravanan, et al.	1	1	3	4	2
**The Influence of Covid-19 Lockdown on Body Mass Index, Depression, Anxiety and Stress among Medical Students.**	Masud, et al	2	2	2	4	3
**Medical students' awareness of COVID-19 against the background of remote learning**	Kuchma, et al	1	1	3	4	2
**P.700 Prevalence of depression in medical students during lockdown in Brazil due to COVID-19 pandemic**	Miskulin, et al	1	1	1	4	1
**The Psychological Impact of the Covid-19 Lockdown on Medical Students of a College in North INdia**	Kumar, et al	1	1	1	4	1
**A survey on anxiety and depression level among South Indian medical students during the COVID 19 pandemic**	Nisha, et al	1	1	1	4	1
**Study of depression, anxiety and stress among first year medical students in Government Medical College, Himachal Pradesh during COVID-19 pandemic.**	Rana, et al	1	1	1	4	1
